# Optimized qRT-PCR Approach for the Detection of Intra- and Extra-Cellular SARS-CoV-2 RNAs

**DOI:** 10.3390/ijms21124396

**Published:** 2020-06-20

**Authors:** Tuna Toptan, Sebastian Hoehl, Sandra Westhaus, Denisa Bojkova, Annemarie Berger, Björn Rotter, Klaus Hoffmeier, Jindrich Cinatl, Sandra Ciesek, Marek Widera

**Affiliations:** 1 Institute of Medical Virology, University Hospital Frankfurt am Main, Goethe University, 60590 Frankfurt am Main, Germany; Tuna.ToptanGrabmair@kgu.de (T.T.); sebastian.hoehl@kgu.de (S.H.); sandra.westhaus@kgu.de (S.W.); denisa.bojkova@kgu.de (D.B.); Annemarie.Berger@kgu.de (A.B.); cinatl@em.uni-frankfurt.de (J.C.J.); sandra.ciesek@kgu.de (S.C.); 2 GenXPro GmbH, Frankfurter Innovationszentrum, Biotechnologie (FIZ), 60438 Frankfurt am Main, Germany; rotter@genxpro.de (B.R.); hoffmeier@genxpro.de (K.H.)

**Keywords:** SARS-CoV-2, COVID-19, qRT-PCR detection, test protocol, coronavirus

## Abstract

The novel coronavirus SARS-CoV-2 is the causative agent of the acute respiratory disease COVID-19, which has become a global concern due to its rapid spread. Meanwhile, increased demand for testing has led to a shortage of reagents and supplies and compromised the performance of diagnostic laboratories in many countries. Both the World Health Organization (WHO) and the Center for Disease Control and Prevention (CDC) recommend multi-step RT-PCR assays using multiple primer and probe pairs, which might complicate the interpretation of the test results, especially for borderline cases. In this study, we describe an alternative RT-PCR approach for the detection of SARS-CoV-2 RNA that can be used for the probe-based detection of clinical isolates in diagnostics as well as in research labs using a low-cost SYBR green method. For the evaluation, we used samples from patients with confirmed SARS-CoV-2 infections and performed RT-PCR assays along with successive dilutions of RNA standards to determine the limit of detection. We identified an M-gene binding primer and probe pair highly suitable for the quantitative detection of SARS-CoV-2 RNA for diagnostic and research purposes.

## 1. Introduction

In early January 2020, the novel severe acute respiratory coronavirus 2 (SARS-CoV-2) was discovered to be the causative agent of the coronavirus disease 2019 (COVID-19). The SARS-CoV-2 outbreak was first detected in late December in Wuhan, China [1] and was recently declared a pandemic by the WHO (WHO, COVID-19 Situation Report—51, published 11 March [2]). We also observed a sharp increase in cases in Germany, and containment measures were imposed to slow the progression. Detecting cases is difficult, as the disease is both very contagious and can be clinically unremarkable in individuals shedding the virus [3,4]. Therefore, the testing of suspected cases is of central importance, but it is also necessary to guide patient care and to apply appropriate hygiene measures in hospitals to restrict nosocomial spread among patients and medical personnel [5]. The pandemic, however, poses unprecedented challenges for institutions conducting the virologic testing, as measures of public health and patient care depend on timely, reliable results.

Quantitative reverse transcriptase polymerase chain reaction (qRT-PCR) is the gold standard in the detection of SARS-CoV-2. Distinct qRT-PCR testing protocols were swiftly established and made publicly available by the WHO [6] and by the Center for Disease Control (CDC) [7].

The protocol published by Corman et al. was designed before the sequence information of the virus isolates was available and recommends a two-step process [6]. In this workflow, the initial screening test is conducted with non-SARS-CoV-2-specific primers binding in the E-gene region coding for the Envelope small membrane protein. The confirmation of the E-gene-positive samples should then be re-tested using primer pairs binding to the coronavirus RNA dependent RNA polymerase (RdRP), which is specific in combination with a probe (P2) but less sensitive [8]. Here, the specificity of the confirmatory test relies on the probe target sequence, which has two mismatches compared to SARS-CoV and contains two wobble positions that most likely hamper the sensitivity. The CDC operates with a distinct protocol targeting three different regions within the N-gene encoding for the viral Nucleoprotein [7]. In both protocols, multiple probes and primers are used in a multi-step PCR workflow, which is laborious and might also complicate the interpretation of the results. In light of the current shortage of reagents, personnel and equipment, a one-step PCR protocol achieving both high sensitivity and specificity would be beneficial for facing the SARS-CoV-2 pandemic. Optimized methods for both diagnostic and the preemptive testing are essential for preventing the worst-case situation.

In this study, we designed an alternative PCR approach specific for the detection of SARS-CoV-2 RNA with primers binding in the M-gene encoding for the viral Membrane protein. This novel primer pair exerts a higher specificity than the E-gene protocol but has significantly better sensitivity when compared with the RdRP-based PCR. As our approach requires fewer reagents and less hands-on time, it could be advantageous for early virus detection, especially in countries where resources are limited.

## 2. Results

### 2.1. Evaluation of Specific PCR Approaches for the Detection of SARS-CoV-2 RNA

**Figure 1:**
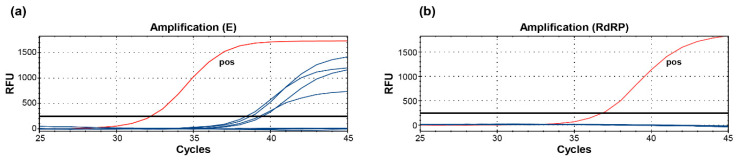
The WHO E-gene PCR for the pre-screening for SARS-CoV-2 using in vitro diagnostic (IVD)-certified test kits (Roche) produces unspecific, template-independent side-products. (**a**) Detection of SARS-CoV-2 false-positive samples (Pat. 20–23) tested with primers targeting the E-gene. (**b**) Confirmatory PCR using SARS-CoV-2 RdRP-specific primer and probe. RFU, relative fluorescence units; *pos*, positive control SARS-CoV-2 positive Pat.1.

In several laboratories, non-specific PCR products in both SARS-CoV-2-negative patient samples and in the non-template control (NTC) using the WHO-recommended SARS-CoV E-gene-specific PCR [6] have been reported [8] (personal communication). During a surveillance pool testing of SARS-CoV-2 using RNA prepared from routine respiratory samples [9], we observed discordant results for E-gene and RdRP-gene qPCR analysis (Supplementary Table S1). We then individually analyzed the pool sets in order to identify samples giving positive signals (Table S1). In total, four out of the 48 (8.33%) tested swab samples showed a positive signal in the E-gene PCR, which, however, could not be confirmed in the RdRP-specific PCR. In order to confirm and further characterize these non-specific products in the negative samples, we carried out additional PCR-based analyses from the four E-gene-positive pharyngeal swab samples (Pat. 20–23, Table S1) with the WHO-recommended qRT-PCR protocol using E (Figure 1a)- and RdRP (Figure 1b)-gene-specific primers (Table 1). As a positive control, we used RNA extracted from a throat swab of one mildly symptomatic passenger (Pat.1) returning from Wuhan after initial quarantine (Figure 1, Table 2), described previously [4]. Again, all four samples showed a positive signal in the E-gene PCR; however, the confirmatory RdRP-gene PCR was negative except for the positive control (Figure 1b). These data indicate that using the two-step WHO PCR protocol might complicate the interpretation of the test results, especially for cases with higher Cq-values. 

Since the RdRP-gene PCR is more specific than the E-gene PCR but is also described as less sensitive [8], we aimed to develop a novel approach using specifically designed primer and probe pairs. For optimal primer design, consensus sequences were aligned to the reference SARS-CoV-2 genome sequence and analyzed for primers binding in the E, N, Orf1, M and S regions (Table 1, Figure S1, Table S3) that would allow SARS-CoV-2 but not SARS-CoV amplification. For experimental confirmation, seven different CoV-2 isolates were propagated and characterized for this study (Table 2). Briefly, patient samples were passaged in Caco-2 cells as described previously [10], and the progeny viruses (FFM1–7) were purified from culture supernatants for subsequent PCR and next generation sequencing (NGS) analysis. In addition, we included RNA from the human SARS-CoV strain Frankfurt 1 (NC_004718) to the analysis. We then compared the PCR performance using serial dilutions; however, the Cq values for the E- and N-gene-specific primers revealed limited linearity and thus were excluded from further analysis (Figure S1a). The S- and M-gene-based PCRs were more sensitive than Orf-1 (Figure S1b) and proved to be suitable for linear SARS-CoV-2 RNA detection, including for samples with low viral loads (~Cq 40), while only exceptionally high SARS-CoV samples were cross-reactive with high Cq values (Figure S1c). Since the M-gene PCR was superior with an approximately Cq 1.73 +/− 0.26 earlier detection (Cq 1.48 +/− 0.15 for SARS-CoV, Figure S1d), we continued with the M-gene PCR and further characterized the limit of detection.

In order to further assess the M-gene PCR’s efficiency, the M- and RdRP-gene-specific PCR primers and probe binding sites were analyzed. The alignment of over 4300 full-length SARS-CoV2 genomes including FFM1–7 isolates revealed single nucleotide polymorphisms (SNPs) in 13 different positions for RdRP (total 0.33%) and eight positions for the M-gene (total 0.61%) within the primer/probe binding sites (Figure S3, Table S4). Among seven, a C/U mismatch within the probe binding site at Position 27,046 of the reference genome was the most common SNP with a 0.43% frequency. At the time of the initial submission of this manuscript, analysis with 165 full-length genomes revealed a 100% identity for both primers, and only one isolate of 165 (0.6%) had a mismatch to the consensus sequence C/U. Beside this particular locus, the M-gene region seems to be less polymorphic compared to the RdRP region, and thus, this may overall result in better PCR efficiency.

**Figure 2:**
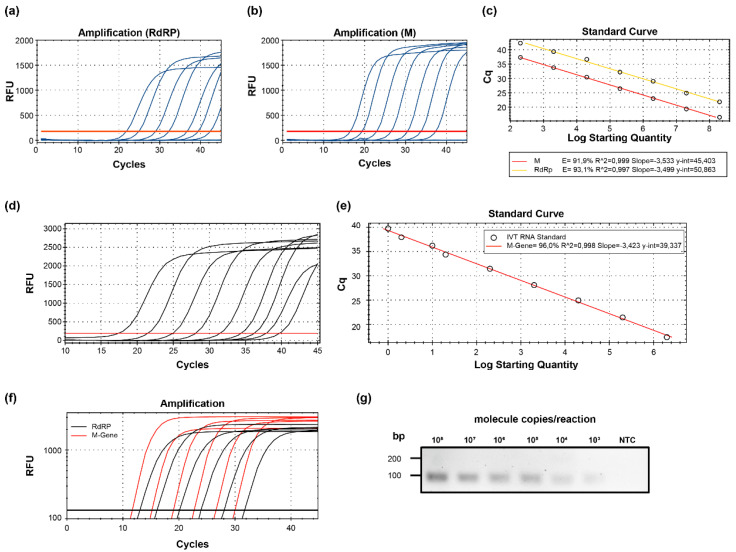
Characteristics of M-gene based qRT-PCR. (**a**) Representative amplification curves of WHO RdRP- and (**b**) SARS-CoV-2 M-gene-specific qRT-PCR using plasmid standard copy numbers. (**c**) Standard curves of both genes determined with plasmid templates. The Cq was plotted against the log starting quantity of the indicated plasmid DNA template. (**d**) Representative amplification curves and (**e**) standard curves of SARS-CoV-2 M-gene-specific qRT-PCR using in vitro-transcribed RNA templates. Log starting quantity (copies/reaction) is indicated and plotted against the Cq. (**f**) Representative amplification curves for SARS-CoV-2 M- and RdRP-gene-specific qRT-PCR using in vitro-transcribed RNA templates harboring both target sequences. (**g**) Distinct M-gene amplicons visualized in a 2% agarose gel. The RNA copy numbers per reaction are indicated on the top. RFU, relative fluorescence units; bp, base pairs; Cq, quantification cycle; NTC, no template control (H_2_O); E, amplification efficiency.

Using plasmid DNA constructs that harbor the conserved SARS-CoV-2 amplicon sequences, we generated standard curves and compared the M-gene PCR (Figure 2b) with the established WHO RdRP-gene PCR approach (Figure 2a). Using the same dilution series of plasmid samples, the M- gene-based method allowed earlier detection (Figure 2c). To determine the analytical sensitivity of the M-gene-based approach, we additionally used in vitro-transcribed RNA standards and tested four replicates to determine the limit of detection (Figure 2d,e,g). To rule out the possibility that different RNA preparations might cause a bias in the comparison, we have also generated an in vitro-transcribed RNA standard that harbored both target sequences. Using an in vitro diagnostic (IVD)-certified test kit (Roche, Basel, Switzerland)*,* we confirmed the M-gene PCR to be more sensitive than RdRP (Figure 2f). SYBR green-based melting curve analysis revealed a melting point at 80 °C for all the samples (Figure S2). To further characterize the capacity of the M-gene PCR to specifically detect high viral loads, we performed SYBR green-based PCR and quantified high loads of virus in cell culture supernatants (Figure S2d).

In conclusion, our M-gene-based qRT-PCR detection of SARS-CoV-2 RNA was at least as specific as the RdRP PCR recommended for confirmation by the WHO but showed a significantly higher sensitivity. Importantly, non-specific signals, as observed in the E-gene PCR, were not detected. 

### 2.2. Detection of SARS-CoV-2 in Clinical and Research Samples Using E-, RdRP-, and M-Gene-Specific Protocols

**Figure 3:**
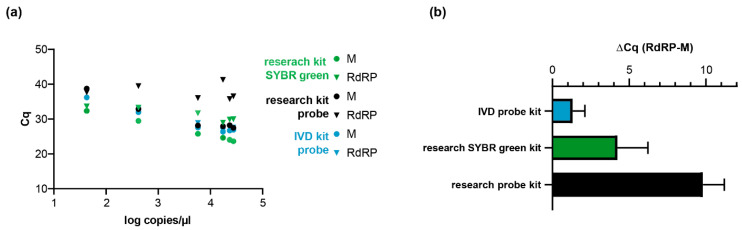
SARS-CoV-2 RNA detection in patient samples detection using M- versus RdRP-specific primers. (**a**) Comparison of M- and RdRP-specific primers and probes measured with the indicated one step qRT-PCR kits. (**b**) Mean values of the Cq difference between RdRP- and M-gene PCR. Cq, quantification cycle. Only samples positive in all assays are shown (*n* = 6).

In order to validate our method, we re-tested clinically relevant samples that had been qualitatively tested as positive for SARS-CoV-2 RNA during routine diagnostics (Table S2). WHO-recommended RdRP primer pairs were used for confirmation. As negative controls, we included eight negative samples (Figure 1, Table S1**)**. As described above, non-specific E-gene amplicons were detected in a test kit-specific manner, possibly due to the reagents used [8]. Therefore, we additionally compared the performance of two research kits (New England Biolabs, Ipswich, MA, USA) and one in vitro diagnostic (IVD) certified test kit (Roche Viral Multiplex RNA Kit) using M-gene and RdRP-gene primers. Overall, we observed lower Cq values with all three kits for the M-gene when compared to the RdRP-gene PCR (Figure 3. Using the Luna OneStep Probe kit (Luna Universal Probe One-Step RT-qPCR Kit, NEB), the M-gene PCR was significantly more sensitive with all the tested kits, with a difference of approximately 10 Cq values with the Luna Universal Probe One-Step RT-qPCR Kit in comparison to PCR with the RdRP-specific primer pairs. With the SYBR green-based kit (Luna Universal One-Step RT-qPCR Kit) and Roche IVT kit (LightCycler^®^ Multiplex RNA Virus Master), we observed four and one Cq value difference(s), respectively, confirming that the detection of the M-gene is superior to the RdRP-gene PCR. 

**Figure 4:**
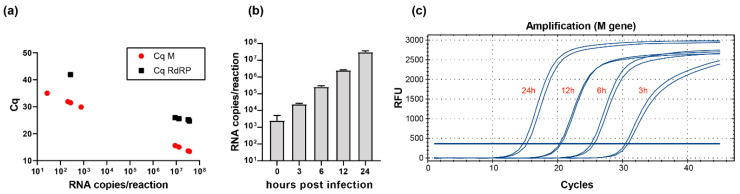
Detection of intracellular SARS-CoV-2 RNAs. (**a**) Vero cells were infected with SARS-CoV-2 isolate FFM1 (MT358638) with high and low MOI, and 24 h post infection, the RNA was subjected to M- and RdRP-gene-specific one-step probe qRT-PCR analysis (Luna, NEB). Intracellular copy numbers (**b**) and representative amplification curves (**c**) of SARS-CoV-2 isolate FFM1-infected Caco-2 cells determined with the M-gene primer pairs and probe. Total cellular RNA including viral RNA was harvested at the indicated time points and subjected to M-gene specific qRT-PCR analysis.

To further evaluate whether the newly developed method is also suitable for the detection of intracellular virus RNA, we infected Vero and Caco-2 cells with SARS-CoV-2 isolate FFM1 (MT358638). Using M-gene PCR, we were able to detect very low copy numbers of viral RNA in infected Vero cells, while RdRP-gene PCR was limited in sensitivity (Figure 4a). Furthermore, we infected Caco-2 cells, generated an intracellular replication curve, and monitored viral replication and genome copy numbers, respectively. Viral RNA was detectable at the time points of 3, 6, 12 and 24 h post infection, with a linear trend (Figure 4b,c).

In conclusion, non-specific PCR products and limited sensitivity pose a major problem when using the WHO protocol for SARS-CoV-2 detection and could lead to numerous unnecessary confirmation tests. Our newly developed PCR protocol is suitable for saving time and resources since pre-screening is no longer necessary. Thus, this approach might be used as a cost-effective alternative to the E- and RdRP-based protocol for research purposes and for diagnostics in resource-limited settings.

## 3. Discussion

Countermeasures against COVID-19 depend on testing with the highest sensitivity and specificity possible. We and others [8] (personal communication) determined certain drawbacks of the two-step PCR protocol recommended by the WHO, including unspecific signals for E-gene PCR arising due to a combination of several factors including primer dimers, the unspecific binding of the primers and probes, the RT-PCR kit and thermocycler-dependent differences. In commercially available kits, primers and probes, as well as the buffers and enzyme quantities and properties, have been matched to one another to reduce non-specific amplification [8]. In times of increasing reagent shortages, however, a simple protocol that can be quickly adapted with universal test kits (including research kits) and is easy to evaluate would be beneficial.

**Figure 5:**
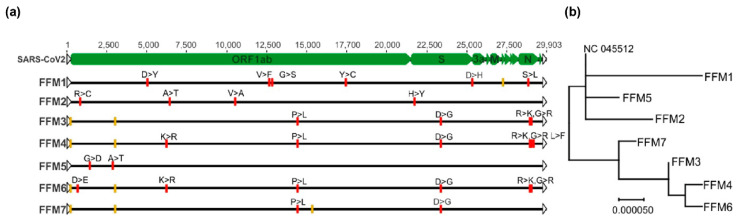
SARS-CoV-2 sequences are highly conserved in terms of the M-gene. (**a**) SARS-CoV-2 isolates FFM1–7 described in this study were mapped to the NC_045512 (SARS-CoV-2) reference genome using the Geneious Prime software. Single nucleotide polymorphisms and their corresponding positions are indicated as colored boxes. Amino acid substitutions are shown above red boxes. Silent mutations are indicated in yellow boxes. (**b**) Phylogenetic tree of SARS-CoV-2 isolates, inferred by using the Maximum Likelihood method and Tamura–Nei model using the MEGA-X software. The tree is drawn to scale, with branch lengths representing the number of substitutions per site.

In this study, we evaluated several primer and probe pairs designed in silico and identified M-gene-specific primers and probes (Table 1) to be highly versatile for the detection of SARS-CoV-2 RNA in a large selection of samples. We analyzed samples from patients presenting with different symptoms, viral loads and degrees of infection (Table 2, Tables S2 and S3) and compared isolates with varying demographic characteristics originating from several infection clusters (Table 2). Interestingly, via sequence comparison based on phylogenetic analysis, we were able to show that isolate FFM5, with unknown origin, shows a high degree of relation to the FFM1 isolate from China, indicating the same origin (Figure 5). Nevertheless, the SARS-CoV-2 M-gene PCR was able to detect SARS-CoV-2 from all clusters.

In a direct comparison of the RdRP PCR recommended by the WHO and the M-Gene PCR developed in this study, the M-Gene PCR was superior in terms of sensitivity using multiple test kits. Since we used primers that specifically bind SARS-CoV-2 RNA, this was to be expected. The reduced sensitivity of the WHO-recommended RdRP-PCR may be explained by the presence of multiple wobble nucleotides (Table 1) in the primer and probe sequences [6]. Sequence alignment analysis showed that M-gene region is less polymorphic; however, the commonly detected C→U SNP at genome position 27,046 may influence the PCR’s efficiency when testing for the isolates carrying this mutation. The M-gene PCR with 16 clinical samples including the sequenced virus isolates FFM1–7 consistently showed a superior efficiency when compared to the RdRP-gene PCR. It is possible that similar to the FFM1–7 isolates, the other clinical samples tested here did not carry this mutation or this SNP does not pose any considerable limitation. Nevertheless, using a Y (C or T) wobble nucleotide or extending the probe-binding sequence may overcome this problem. In a situation of crisis, where the availability of reagents, equipment and labor of qualified personnel are limited, a reliable and convenient one-step PCR protocol with optimum sensitivity would be beneficial.

Our comparison analysis for M- and RdRP-gene PCR was consistent and reproducible for detecting RNA from progeny virus particles and also intracellular viral mRNAs extracted from infected Caco-2 and Vero cells. Full-length SARS-CoV-2 genomic RNA serves as a template for replication and transcription. In addition to the genomic RNA, nine subgenomic RNAs—including the structural protein S, E, M and N coding regions—are generated by discontinuous transcription and leader sequence fusion during negative-strand synthesis. Even if some of the subgenomic viral mRNAs (ORF6, ORF7a, ORF7b, ORF8, N and ORF10) do not harbor the M-gene target sequence, along with the N and S genes, M transcripts are abundantly expressed and may therefore serve as a more sensitive target for diagnostic detection when compared to the RdRP and E genes [11]. We have also shown that the method is also suitable for inexpensive SYBR green-based PCR assays (Figure S2d). Thus, M-gene PCR allows the quantification of very low and very high viral loads and might be used as a cost-effective alternative to the E- and RdRP-based protocol for research purposes and for diagnostics in resource-limited settings.

## 4. Materials and Methods

### 4.1. Cell Culture and Virus Preparation

Caco-2 (human colon carcinoma) and Vero cells (African Green monkey kidney) were cultured in Minimum Essential Medium (MEM) supplemented with 10% fetal calf serum (FCS), 100 IU/mL of penicillin and 100 g/mL of streptomycin. All culture reagents were purchased from Sigma (St. Louis, MO, USA). The Caco-2 cells were originally obtained from DSMZ (Braunschweig, Germany, no.: ACC 169), differentiated by serial passaging and selected for high permissiveness to virus infection. Vero cells were obtained from ATCC (Manassas, VA, USA, ATCC-CCL81). The SARS-CoV-2 isolate Frankfurt 1 (SARS-CoV-2 FFM1) was obtained from the throat swab of a patient diagnosed with SARS-CoV-2, hospitalized in the isolation unit of Frankfurt University Hospital (Germany) [4]. The SARS-CoV isolate FFM-1 was isolated from a patient diagnosed with a SARS-CoV infection in 2003 at Frankfurt University Hospital [12]. SARS-CoV-2 and SARS-CoV were propagated in Caco-2 and Vero cells respectively in MEM supplemented with 1% FCS. Virus stocks that were stored at −80 °C. Viral titers were determined by TCID_50_ or qRT-PCR. In accordance with the decision of the Committee on Biological Agents (ABAS) and Central Committee for Biological Safety (ZKBS), all the work involving infectious SARS-CoV-2 and SARS-CoV was performed under biosafety level 3 (BSL-3) conditions in a BSL-3 facility. For intracellular RNA testing, 1 × 10^5^ Vero cells were seeded per well in a 12-well plate. Cells were inoculated with SARS-CoV-2 or SARS-CoV suspensions at an MOI of 0.1 for one hour at 37 °C, under 5% CO_2_. Subsequently, cells were rinsed with PBS, fed with fresh medium and incubated for another 7 days before harvesting for RNA extraction.

For time point analysis, Caco-2 cells were infected with SARS-CoV-2 (0.01 MOI) in MEM supplemented with 1% FCS, at 37 °C under 5% CO_2_. Cells were harvested for RNA extraction at 0, 3, 6, 12 and 24 h post infection using TRI Reagent (Sigma, St. Louis, MO, USA). Following chloroform extraction and isopropanol precipitation, RNA was dissolved in nuclease-free water and treated with DNase I (Qiagen, Hilden, Germany) for 15 min at 37 °C. DNase I-treated RNA was subjected to RT-PCR analysis.

### 4.2. Quantification of SARS-CoV-2 RNA

SARS-CoV-2 RNA from cell culture supernatant samples was isolated using AVL buffer and the QIAamp Viral RNA Kit (Qiagen) according to the manufacturer’s instructions. Intracellular RNAs were isolated using the RNeasy Mini Kit (Qiagen) as described by the manufacturer. RNA was subjected to OneStep qRT-PCR analysis using the Luna Universal One-Step RT-qPCR Kit (New England Biolabs) or Luna Universal Probe One-Step RT-qPCR Kit (New England Biolabs) or LightCycler^®^ Multiplex RNA Virus Master (Roche) using the CFX96 Real-Time System, C1000 Touch Thermal Cycler. The primer pairs for the E-, S- and M-gene-specific PCRs were used in equimolar concentrations (0.4 µM each per reaction). The RdRP primer pairs were used according to Corman et al. [6] with 0.6 µM and 0.8 µM concentrations of the forward and reverse primers, respectively. The cycling conditions were used according to the manufacturer’s instructions. Briefly, for SYBR green-and probe-based Luna Universal One-Step RT-qPCR Kits, 2 µL of RNA was subjected to a reverse transcription reaction in a reaction volume of 20 µL, performed at 55 °C for 10 min. Initial denaturation was performed for 1 min at 95 °C, followed by 45 cycles of denaturation for 10 s and extension for 30 s at 60 °C. Melt curve analysis (SYBR green) was performed from 65–95 °C with an increment of 0.5 °C each 5 s. For the IVD-approved LightCycler^®^ Multiplex RNA Virus Master (Roche), 5 µL of template RNA in a total reaction volume of 20 µL was used. Reverse transcription was performed at 55 °C for 10 min. Initial denaturation was induced for 30 s at 95 °C, followed by 45 cycles of denaturation for 5 s at 95 °C, and extension for 30 s at 60 °C, and a final cool-down to 40 °C for 30 s. The PCR runs were analyzed with the Bio-Rad CFX Manager software, version 3.1 (Bio Rad Laboratories, Hercules, CA, USA).

### 4.3. Primer Design

For primer design and validation, 165 available SARS-CoV-2 full-length sequences were aligned using NCBI Virus Variation Resource [13], and a consensus was made with a 99% identity cutoff. The Primer3 and Geneious Prime^®^ software, version 2020.0.5 (Biomatters Ltd., Auckland, New Zealand) were used to design primers and probes matching the consensus sequence. Oligo characteristics were calculated using a modified version of Primer3 2.3.7 (integrated Plugin in Geneious Prime software). Positions within the SARS-CoV-2 genome were determined according to accession number MN908947. Illustrations were made with the Geneious Prime software.

### 4.4. Generation of DNA and RNA Standard Curves

Standard curves were created using plasmid DNA harboring the corresponding amplicon target sequence according to GenBank Accession number NC_045512. For in vitro transcription, pCR2.1-based plasmid DNA (pCR2.1 SARS-CoV-2 M (475-574)) was linearized with BamHI. In vitro transcription was carried out using the HiScribeT7 High Yield kit (NEB) according to the manufacturer’s instructions. For the generation of the dual target RNA, the RdRP target-sequence-containing fragment was extracted from the plasmid pEX-A128-RdRP [10] using EcoRI and cloned into the plasmid pCR2.1 SARS-CoV-2 M (475-574), linearized with EcoRI, resulting in pCR2.1 SARS-CoV-2 M-RdRP. In vitro transcription was performed as described after linearization with HindIII. Absorbance-based measurement of the RNA yield was performed using the Genesys 10S UV-Vis Spectrophotometer (Thermo Scientific, Waltham, MA, USA).

### 4.5. Illumina NGS Sequencing of SARS-CoV-2 Isolates

Caco-2 cells were infected with different viral isolates (FFM1-FFM7) at an MOI of 0.01. Cell culture supernatant was harvested at 48 h after infection then precleared at 2000× *g* for 10 min at room temperature. Virus particle-containing supernatant (20 mL) was overlaid on 25% sucrose in PBS and centrifuged at 26,000 rpm for 3 h at 4 °C using an SW28 rotor (Beckman Coulter, Brea, CA, USA). Pellets were dissolved in 500 µL of sterile PBS overnight at 4 °C. One third of the purified virions was used for RNA extraction using the Macherey Nagel Virus RNA isolation kit (Macherey Nagel, Düren, Germany) without carrier RNA, according to the manufacturer’s recommendations. The libraries were prepared and sequenced by GenXPro GmbH, Frankfurt a. M., Germany. Briefly, quality was checked on a LabChip GXII platform, and RNA-seq libraries were prepared using the NEBNext Ultra II Directional RNA-Seq kit according to the manufacturer’s recommendations. Sequencing was performed on an Illumina NextSeq 500 platform with 75 cycles. The raw sequencing reads were trimmed for adaptors and for a minimum quality of 30 and mapped to NC_045512 using Geneious mapper with default settings. The full-length sequences for each isolate can be retrieved using Genbank accession numbers MT358638–MT358643. The phylogenetic tree was inferred by using the Maximum Likelihood method and Tamura–Nei model [14] and conducted by the MEGA-X software [15].

## Figures and Tables

**Table 1:** Oligonucleotides used for the detection of SARS-CoV-2 RNA.

Oligo Name	Oligonucleotide Sequences (5’ to 3’)	Position within the SARS-CoV-2 Genome	Length (nt)	Deg.	Mism.	Ref.
E_Sarbeco_F1	ACAGGTACGTTAATAGTTAATAGCGT	26,269–26,294	26			[4]
E_Sarbeco_R2	ATATTGCAGCAGTACGCACACA	26,360–26,381	22			
E_Sarbeco_P1	6-Fam ACACTAGCCATCCTTACTGCGCTTCG BBQ1	26,332–26,357	26			
RdRP_SARSr-F2	GTGARATGGTCATGTGTGGCGG	15,431–15,452	22	2		
RdRP_SARSr-R1	CARATGTTAAASACACTATTAGCATA	15,505–15,530	26	4	1 ^1^	
RdRP_SARSr-P2	6-Fam CAGGTGGAACCTCATCAGGAGATGC BBQ1	15,470–15,494	25			
M-475-F	TGTGACATCAAGGACCTGCC	26,997–27,016	20			
M-574-R	CTGAGTCACCTGCTACACGC	27,077–27,096	20			
M-507-P	6-Fam TGTTGCTACATCACGAACGC BHQ1	27,029–27,048	20			

^1^ Mismatch (Mism.) to SARS-CoV-2 is covered by a degenerate position (S). Degeneracy (Deg.). IUPAC codes: S = G or C; R = A or G

**Table 2:** Compilation of patient samples and isolated viral isolates in this study.

Patient	Virus Isolate	Material	Age/Gender	Cluster of Infection	Symptoms	Accession
Pat.1	FFM1	throat swab	44/female	Hubei, China	dry cough, sore throat	https://www.ncbi.nlm.nih.gov/nuccore/MT358638
Pat.2	FFM2	throat swab	58/male	Hubei, China	asymptomatic	https://www.ncbi.nlm.nih.gov/nuccore/MT539726
Pat.12	FFM3	nasopharyngeal swab	30/male	Italy, Austria	diarrhea, rhinitis	https://www.ncbi.nlm.nih.gov/nuccore/MT358639
Pat.11	FFM4	throat swab	32/male	Italy	cough, muscle ache, fever	https://www.ncbi.nlm.nih.gov/nuccore/MT358640
Pat.15	FFM5	respiratory swab	42/female	Germany	unknown	https://www.ncbi.nlm.nih.gov/nuccore/MT358641
Pat.14	FFM6	nasopharyngeal swab	27/male	Italy	cough, rhinitis, headache, muscle ache, abdominal pain	https://www.ncbi.nlm.nih.gov/nuccore/MT358642
Pat.7	FFM7	throat swab	61/male	Israel	sore throat	https://www.ncbi.nlm.nih.gov/nuccore/MT358643

## Supplementary Materials

Supplementary materials can be found at https://www.mdpi.com/1422-0067/21/12/4396/s1.

## Abbreviations

SARS-CoV-2	severe acute respiratory syndrome coronavirus 2
Cq	quantification cycle
COVID-19	coronavirus disease 2019
bp	base pairs
RFU	relative fluorescence units
IVD	in vitro diagnostic
