# Supplementary material for: Optimized qRT-PCR Approach for the Detection of Intra- and Extra-Cellular SARS-CoV-2 RNAs
Source: Int J Mol Sci. 2020 Jun 20;21(12):4396. doi: 10.3390/ijms21124396 (PMC7352576; doi:10.3390/ijms21124396)
Supplement: Supplementary file 1 [file ijms-21-04396-s001.pdf]

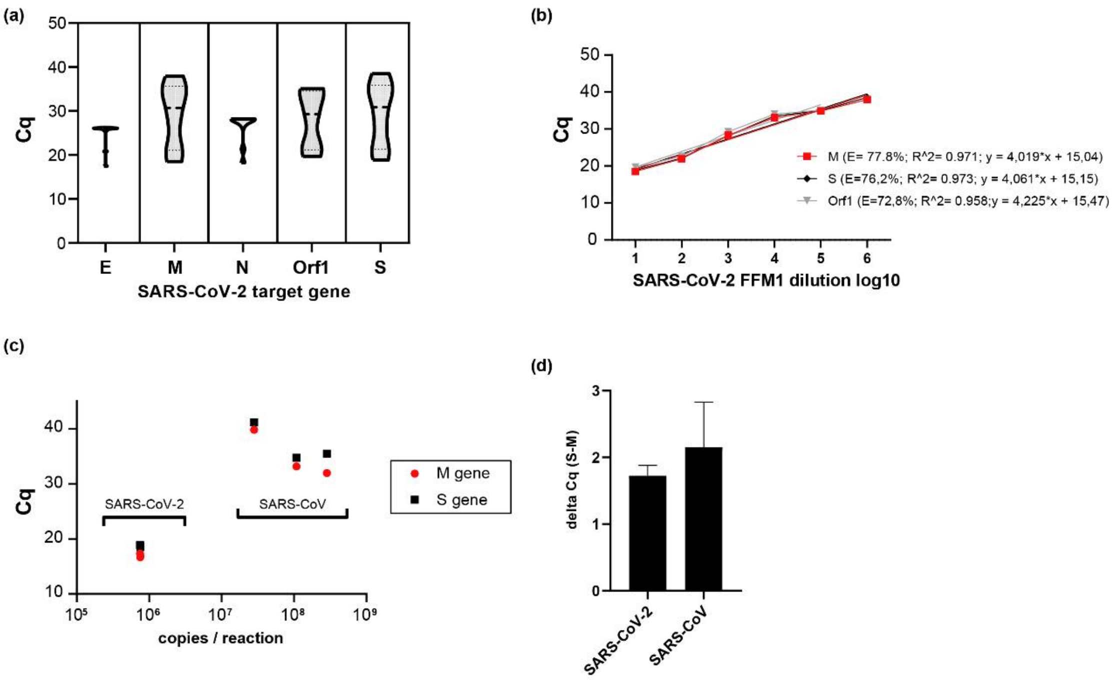

Supplementary Figure 1. Evaluation of SARS-CoV-gene-specific qRT-PCR. (**a**) Violin plots showing Cq distributions of successive RNA dilutions of SARS-CoV-2 isolate FFM1 (MT358638). (**b**) Linearity of M-, Orf1- and S-gene qRT-PCRs. Amplification curves of the dilution series were used for the evaluation of S-and M-gene specific qRT-PCR. (**c**) Detection of SARS-CoV Frankfurt 1 (NC_004718) and SARS-CoV-2 FFM1 (MT358638) strain RNA using M- and S-gene specific primer and probe pairs (Supplementary Table 3). (**d**) Comparison of Cq values obtained with S- or M-specific primes (n = 3). Error bars indicate standard deviation. RFU, relative fluorescence units; bp, base pairs; Cq, quantification cycle.

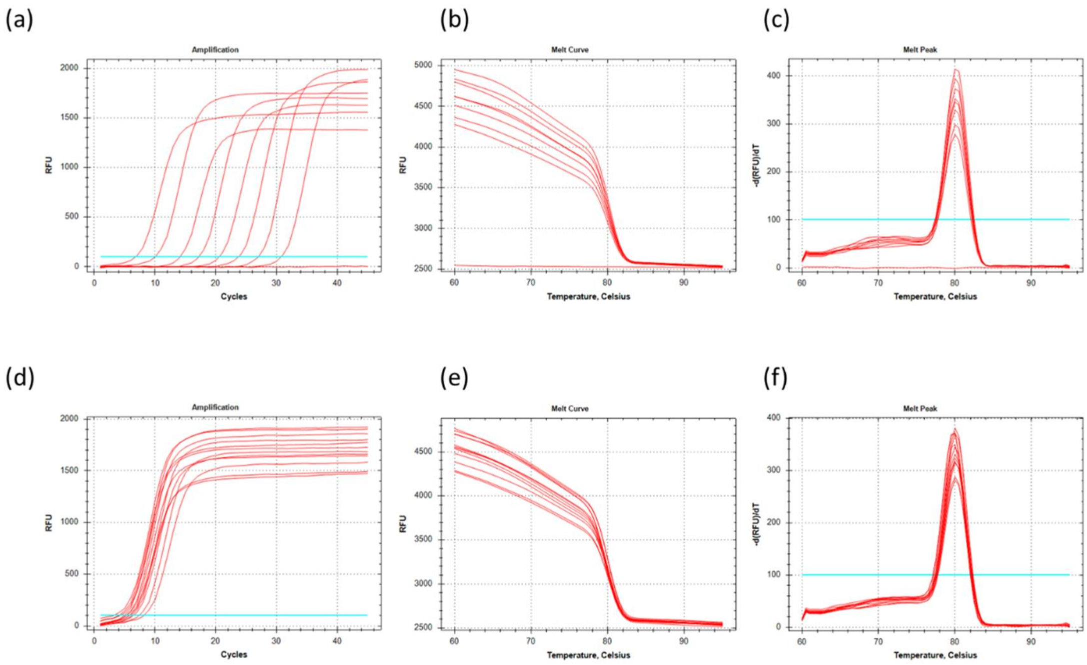

Supplementary Figure 2: Detection of SARS-CoV-2 RNA with M-gene-specific PCR primer and probe pairs using SYBR green. (**a**) Amplification curve of in vitro-transcribed RNA-standards and concentrated virus supernatants (**d**). (**b** and **e**) melt curve analysis and (**c** and **f**) melt peaks of M-gene amplicons. RFU, relative fluorescence units; bp, base pairs; Cq, quantification cycle.

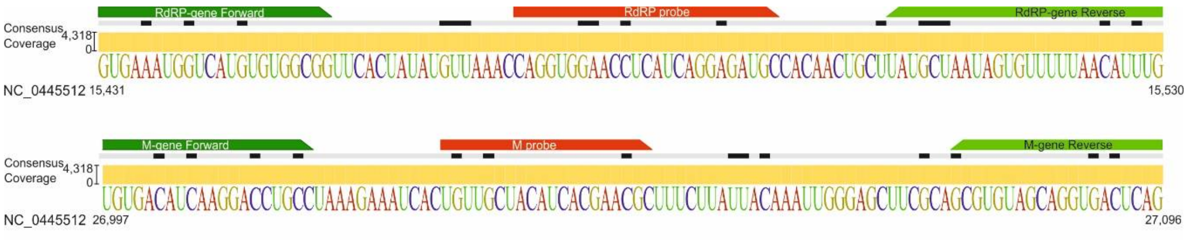

Supplementary Figure 3. The sequence alignment of 4318 SARS-CoV-2 full-length genomes. Mapping of the genomes to the reference sequence was performed using the Geneious mapper program. Primer binding sites of RdRP- and M-gene primers (dark and light green arrows) and probes (red arrows) are indicated. Black bars show the number and position of mismatches found in 4318 full-length SARS-CoV-2 genomes on the reference sequence NC_0445512. Graphical representation of the sequence conservation of nucleotide sequences is depicted as sequence logos. The relative sizes of the letters illustrate their frequency in the compared sequences. The heights of the letters are measures of the information content of the positions in bits.

Supplementary Table 1. Compilation of patient samples used for the pool-testing with diagnostic-approved kits targeting SARS-CoV-2 genes E and RdRp.

Pool-Test Group	Patient No.	E Gene PCR	RdRp Gene PCR
POOL1	Pat.24	negative	negative
	Pat.25	negative	negative
	Pat.26	negative	negative
	Pat.27	negative	negative
	Pat.28	negative	negative
	Pat.29	negative	negative
	Pat.30	negative	negative
	Pat.31	negative	negative
	Pat.32	negative	negative
	Pat.33	negative	negative
POOL2	Pat.22	positive	negative
	Pat.18	negative	negative
	Pat.19	negative	negative
	Pat.23	positive	negative
	Pat.21	positive	negative
	Pat.34	negative	negative
	Pat.35	negative	negative
	Pat.36	negative	negative
	Pat.37	negative	negative
	Pat.38	negative	negative
POOL3	Pat.39	negative	negative
	Pat.17	negative	negative
	Pat.16	negative	negative
	Pat.40	negative	negative
	Pat.20	positive	negative
	Pat.41	negative	negative
	Pat.42	negative	negative
	Pat.43	negative	negative
	Pat.44	negative	negative
	Pat.45	negative	negative
POOL4	Pat.46	negative	negative
	Pat.47	negative	negative
	Pat.48	negative	negative
	Pat.49	negative	negative
	Pat.50	negative	negative
	Pat.51	negative	negative
	Pat.52	negative	negative
	Pat.53	negative	negative
	Pat.54	negative	negative
	Pat.55	negative	negative
POOL5	Pat.56	negative	negative
	Pat.57	negative	negative
	Pat.58	negative	negative
	Pat.59	negative	negative
	Pat.60	negative	negative
	Pat.61	negative	negative
	Pat.62	negative	negative
	Pat.63	negative	negative

Supplementary Table 2. Compilation of patient samples used for validation of M-gene PCR in a clinically relevant setting.

Patient No.	Material	Age/Gender	Cluster of Infection	Symptoms, Comorbidity
Pat.1	Throat swab	44/f	Hubei, China	Dry cough, sore throat
Pat.2	Throat swab	58/m	Hubei, China	Asymptomatic
Pat.3	Throat swab	59/m	Israel	Rhinitis, cough, back ache
Pat.4	Throat swab	52/f	Israel	Cough, pharyngitis, fever
Pat.5	Throat swab	55/m	Israel	Cough
Pat.6	Throat swab	64/m	Israel	Asymptomatic
Pat.7	Throat swab	61/m	Israel	sore throat
Pat.8	Throat swab	40/f	Israel	Mild pharyngitis
Pat.9	Throat swab	54/f	Israel	Asymptomatic
Pat.10	Nasopharyngeal swab	43/m	Unknown	Cough
Pat.11	Stool	32/m	Italy	Cough, muscle ache, fever
Pat.12	Nasopharyngeal swab	30/m	Italy, Austria	Diarrhea, sore throat, rhinitis
Pat.13	Respiratory swab	37/f	Unknown	Cough, fever, muscle ache, fatigue
Pat.14	Throat swab	27/m	Italy	Cough, rhinitis, headache, muscle ache, abdominal pain
Pat.15	Respiratory swab	42/f	Germany	unknown
Pat.16	Bronchoalveolar lavage	13/m	SARS-CoV-2 neg.	Non-Hodgkin Lymphoma, RSV-Pneumonia, ARDS
Pat.17	Throat swab	5/m	SARS-CoV-2 neg.	SCT
Pat.18	Throat swab	58/m	SARS-CoV-2 neg.	SCT
Pat.19	Throat swab	70/m	SARS-CoV-2 neg.	Leukemia, SCT
Pat.20	Throat swab	54/m	SARS-CoV-2 neg.	Fever, HIV(+)
Pat.21	Throat swab	64/m	SARS-CoV-2 neg.	SCT
Pat.22	Throat swab	76/m	SARS-CoV-2 neg.	SCT
Pat.23	Throat swab	57/m	SARS-CoV-2 neg.	SCT

f: female; m: male; neg.: negative; SCT: stem cell transplantation; RSV: respiratory syncytial virus; ARDS: acute respiratory distress syndrome.

Supplementary Table 3. Primers used for Supplementary Figure 1.

Oligo Name	Oligonucleotide Sequences (5′to 3′)	Position within the SARS-CoV-2 Genome	Length (nt)	Tm (°C)	%GC
SARS-CoV-2 Probe Orf1ab	6-Fam-ACGTCCAACTCAGTTTGCCT-BBQ1	303–322	20	50.0	59.8
SARS-CoV-2 Orf1ab-gene R	GTCTCCAAAGCCACGTACGA	364–345	20	55.0	60.0
SARS-CoV-2 Orf1ab-gene F	GATGGAGAGCCTTGTCCCTG	265–284	20	60.0	59.8
SARS-CoV-2 Probe-N	6-Fam-CGCATTGGCATGGAAGTCAC-BBQ1	29,228–29,247	20	55.0	60.2
SARS-CoV-2 N-gene R	TGTAGGTCAACCACGTTCCC	29,273–29,254	20	55.0	59.6
SARS-CoV-2 N-gene F	TGGCCGCAAATTGCACAATT	29,174–29,193	20	45.0	60.3
SARS-CoV-2 Probe E	6-Fam-TGCTTTCGTGGTATTCTTGCT-BBQ1	26,307–26,327	21	42.9	58.2
SARS-CoV-2 E-gene R	GCGCAGTAAGGATGGCTAGT	26,353–26,334	20	55.0	59.9
SARS-CoV-2 E-gene F	TTCGTTTCGGAAGAGACAGGT	26,254–26,274	21	47.6	59.3
SARS-CoV-2 Probe S	6-Fam-ACTTACTCCTACTTGGCGTGT-BHQ1	23,446–23,466	21	58.8	47.6
SARS-CoV-2 S-gene F	AAACAGCCTGCACGTGTTTG	23,492–23,511	20	60.2	50
SARS-CoV-2 S-gene R	GCACAGAAGTCCCTGTTGCT	23,412–23,431	20	60.5	55

Supplementary Table 4. Single nucleotide polymorphism positions and frequencies for RdRP- and M-gene primer/probe binding sites extracted from sequence alignment of over 4300 full-length SARS-CoV-2 genomes.

Gene	Primer/Probe	Position (nuc)	Mismatch	# Genomes with Mismatch	% Mismatch	Aligned CoV-2 Genomes
**RdRP**	Forward	15,435	A>G	4	0.15	4318
		15,439	G>K	1		
		15,444	G>K	2		
		15,444	G>T	1		
	Probe	15,476	G>K	1	0.09	
		15,477	A>G	1		
		15,478	A>G	1		
		15,480	C>U	1		
		15,489	A>R	1		
	Reverse	15,508	G>K	1	0.09	
		15,509	C>Y	3		
		15,510	U>W	1		
		15,525	C>U	1		
		15,528	U>Y	1		
**M**	Forward	27,002	C>Y	1	0.14	
		27,005	C>U	4		
		27,011	C>U	1		
		27,015	C>Y	1		
	Probe	27,033	G>K	4	0.43	
		27,046	C>U	16		
		27,046	C>Y	1		
	Reverse	27,090	G>A	1	0.04	
		27,092	C>U	1		

K: T or G; R: A or G; W: A or T; Y: C orT.
